# Stem cells therapy in acute myocardial infarction: a new era?

**DOI:** 10.1007/s10238-021-00682-3

**Published:** 2021-01-23

**Authors:** R. G. Carbone, A. Monselise, G. Bottino, S. Negrini, F. Puppo

**Affiliations:** 1 Department of Internal Medicine, University of Genoa, Genoa, Italy; 2 Tel Aviv University, Tel Aviv, Israel

**Keywords:** Stem cells, Bone marrow cells, Acute myocardial infarction, Transplantation, Clinical trials

## Abstract

Stem cells transplantation after acute myocardial infarction (AMI) has been claimed to restore cardiac function. However, this therapy is still restricted to experimental studies and clinical trials. Early un-blinded studies suggested a benefit from stem cell therapy following AMI. More recent blinded randomized trials have produced mixed results and, notably, the last largest pan-European clinical trial showed the inconclusive results. Furthermore, mechanisms of potential benefit remain uncertain. This review analytically evaluates 34 blinded and un-blinded clinical trials comprising 3142 patients and is aimed to: (1) identify the pros and cons of stem cell therapy up to a 6-month follow-up after AMI comparing benefit or no effectiveness reported in clinical trials; (2) provide useful information for planning future clinical programs of cardiac stem cell therapy.

## Introduction

Transplantation of stem cells by intracoronary infusion after acute myocardial infarction (AMI) represents a novel therapeutic procedure that has been claimed to restore cardiac function. However, the mechanisms underlying its potential efficacy remain unclear. It has been assumed that apoptosis of transplanted cells modulates local immune reactivity deactivating macrophages and dendritic cells and stimulating regulatory T lymphocytes. These phenomena lead to repressed myocardial cells apoptosis thus improving cardiac outcome [1]. Further mechanisms of repair induced by stem cells that have been demonstrated both in experimental models and humans are related to the self-regeneration properties and plasticity of cardiac tissue including: (1) direct cell differentiation from mononuclear cells to cardiac myocytes [2]; (2) cytokine-induced growth of residual viable myocytes [3, 4]; (3) stimulation of resident cardiac stem cells [5]; (4) induction of cell fusion between transplanted stem cells and resident myocytes [6]; and (5) interactions between endothelial cells and cardiomyocytes [7]. Collectively, these mechanisms may lead to a significant increase of perfusion indices and of quality of life.

This article is a systematic review evaluating 34 blinded and un-blinded clinical trials comprising 3142 patients with AMI and is aimed to: (1) identify the pros and cons of stem cell therapy up to a 6-month follow-up after AMI; (2) provide useful information for planning future clinical programs of cardiac stem cell therapy.

## Methods

In order to outline the total number of publications on blinded and un-blinded clinical trials on stem cell therapy in the field of AMI from 2000 to 2020, the search terms “stem cells”, “bone marrow cells”, “acute myocardial infarction”, “transplantation” and “clinical trials” were entered in the search field of the National Library of Medicine database for clinical trials (www.clinicaltrials.gov). A total of 95 studies were registered worldwide, among them 40 were conducted in Europe and 4 in Italy. Phase 1, 2, 3 and 4 studies were 28%, 57%, 14% and 1%, respectively. Observational and randomized studies were 22% and 78%, respectively. Clinical setting of enrolled patients was acute coronary syndrome in 43 studies (46%), heart failure in 29 studies (30%) and chronic heart disease in 23 studies (24%). Among them 34 were blinded and un-blinded clinical trials in AMI patients. In these trials, transplantation of bone marrow-derived cells (BMC) or circulating progenitor cells (CPC), granulocyte-colony stimulating factor (G-CSF) mobilized peripheral blood stem cells (PSC), mesenchymal cells (MSC) and allogeneic cardiac stem cells was utilized in 25 (73.5%), 6 (17.5%), 2 (6%) and 1 (3%) of cases, respectively. Other cell types were used for transplantation in a minority of studies including endothelial precursor cells in 16%, myoblasts in 2% and adipoblasts in 1% of cases, respectively.

## End points

- *Primary* compare per cent increase in mean left ventricular ejection fraction (LFEV) evaluated up to 6 months after stem cells transplantation in AMI.
- *Secondary* compare trials showing benefit or no effectiveness to improve planning of future clinical trials.

## PRO: Trials showing benefit of stem cells therapy in AMI

Reports published in 2002 and 2003 showed for the first time that selective intracoronary transplantation of autologous BMC could be useful in myocardial regeneration and neovascularization beneficially affecting post infarction remodelling processes [8–10].

Subsequent studies confirmed that SC therapy induces: (1) metabolic regeneration of infarcted area and chronic myocardial avital tissue with maximum oxygen uptake increase; (2) improvement in myocardial blood perfusion in the ischaemic region; and (3) durable therapeutic effect and increased exercise capacity in patients with end-stage ischaemic heart disease [11–15].

In BOOST trial, 60 patients with AMI who had undergone percutaneous coronary intervention (PCI) were randomly assigned to BMC harvest and intracoronary infusion into the infarct-related artery or to a control group [16]. The end point was mean LVEF change from baseline to 6-month follow-up. LVEF was significantly greater in the BMC-infused group than in the control group (6.7 versus 0.7 percentage points, *p* = 0.0026). The combination with G-CSF therapy and intracoronary PSC infusion further improved cardiac function promoting angiogenesis. However, the occurrence of restenosis represented a serious side effect of this procedure [16]. The transplantation of PSC after G-CSF mobilization (FIRSTLINE-AMI) reduced this complication offering an effective strategy for preservation of myocardium and prevention of remodelling without evidence of restenosis [17]. The potential value of G-CSF in comparison with the combination of G-CSF and PSC was also assessed in the MAGIC trial [18]. Twenty-seven patients with AMI undergoing PCI were randomly assigned to PSC mobilization with G-CSF followed by stem cell apheresis and intracoronary reinfusion, to G-CSF alone, or to a control group. Six-month follow-up data in 10 of the study patients demonstrated an improvement in LVEF with PSC infusion (from 48.7 to 55.1%) but not with G-CSF alone. PSC infusion also increased treadmill exercise time and reduced the size of the myocardial perfusion defect. However, administration of G-CSF was associated with an unexpectedly high rate of in-stent restenosis of the culprit lesion. The STEM-AMI study recently confirmed that early administration of G-CSF and PSC exerted a beneficial effect in patients with left ventricular dysfunction after ST-segment-elevation myocardial infarction in terms of global systolic function, adverse remodelling, scar size and myocardial strain [19].

In addition, a randomized controlled study demonstrated that mesenchymal stem cells infusion leads to improvement in wall motion and velocity, reduction in ventricular end-systolic and end-diastolic volumes and 14% net increase in ejection fraction [20]. These results were confirmed by Hare et al. [21] and Kim et al. [22] who reported that intracoronary infusions of bone marrow-derived mesenchymal cells improved LVEF in 53 and 26 post-myocardial infarction patients, respectively.

A number of studies validated SC transplantation as a safe and effective procedure in AMI.

The TOPCARE-AMI trial evaluated the effect on coronary blood flow regulation of intracoronary infusion of BMC or CPC into the infarct-related artery in 59 AMI patients. At 4-month follow-up, coronary flow reserve in the infarct artery was markedly increased up to normal in progenitor cell-treated patients compared with only a moderate improvement in placebo group [23].

A following multicentre trial (REPAIR-AMI), including 204 patients who underwent primary PCI after an ST-elevation AMI, was randomly assigned to receive intracoronary infusion of BMC into the infarct-related artery or placebo medium three to six days after AMI. Primary end point, i.e. the absolute increase in LVEF at four months assessed by serial LV angiograms, was significantly higher with active therapy (5.5 versus 3.0% with placebo). Subgroup analyses found that the benefit was limited to patients with baseline LVEF < 49% and to those treated more than 5 days after AMI [24, 25].

The findings of another randomized double-blind controlled trial showed that patients who underwent BMC transfer had a significant per cent reduction in infarct size and a better recovery of regional systolic function that may reflect improved infarct remodelling [26].

Similar findings were noted in a no-blinded observational study of 42 consecutive patients who had a myocardial infarction five months to nine years previously. These patients were compared to a representative control group that did not receive cellular therapy. At three months after intracoronary transplantation of autologous BMC, infarct size was reduced by 30%, LVEF increased by 15% and infarction wall movement velocity increased by 57%. There were no significant changes in the control group [27].

More recent studies including the SCAMI trial that enrolled 42 AMI patients [28] and the PRESERVE-AMI trial [29], the largest study in the USA that enrolled 161 AMI patients with ST-segment elevation, added the evidence that intracoronary infusion of autologous BMC is safe, improves LVEF and reduces risk of death after AMI.

Table 1 summarizes clinical trials showing positive results of stem cells transplantation after AMI.

**Table 1:** Clinical trials reporting benefit of stem cells transplantation after acute myocardial infarction

Authors	Year	Reference	Trials	Cell source	N. patients	Mean% LVEF increase
Strauer BE	2002	[2]	–	BMC	10	2
Assmus B	2002	[3]	TOPCARE-AMI	CPCBMC	119	8.6
Perin EC	2003	[7]	–	BMC	21	3.2
Britten MB	2003	[4]	TOPCARE-AMI	CPCBMC	1513	5
Chen SL	2004	[14]	–	MSC	69	14
Perin EC	2004	[8]	–	BMC	20	10
Schachinger V	2004	[17]	TOPCARE-AMI	CPCBMC	3029	8
Wollert KC	2004	[10]	BOOST	BMC	60	7
Kang HJ	2004	[12]	MAGIC	PSC	27	6.4
Strauer BE	2005	[5]	IACT	BMC	18	15
Ince H	2005	[11]	FIRSTLINE-AMI	PSC	25	6
Janssens S	2006	[20]	–	BMC	67	2.8
Schachinger V	2006	[18]	REPAIR-AMI	BMC	204	7.3
Hare J	2009	[15]	–	MSC	53	6.4
Wohrle J	2013	[22]	SCAMI	BMC	42	4
Assmus B	2014	[19]	REPAIR-AMI	BMC	204	2.5
Duan F	2015	[21]	–	BMC	42	15
Quyyumi AA	2017	[23]	PreSERVE-AMI	BMC	281	2.2
Kim SH	2018	[16]	–	MSC	26	5.4
Achilli F	2019	[13]	STEM-AMI	PSC	161	5.1
Total					1437	

*BMC* bone marrow-derived cells, *CPC* circulating progenitor cells, *MSC* mesenchymal cells, *PSC* G-CSF mobilized peripheral blood stem cells

## CONS: Trials showing no benefit of stem cells therapy in AMI

In contrast with the results reported in the previous paragraph, a series of randomized trials produced mixed and uncertain results on the potential benefit of stem cells therapy in AMI.

In fact, a group of patients with ST elevation AMI undergoing PCI and BMC intracoronary infusion into the infarct-related artery did not show amelioration of LVEF as compared to a control group [26]. However, patients who underwent stem cell transfer had a significant 28% reduction in infarct size and better recovery of regional systolic function, changes that may reflect improved infarct remodelling [26].

In the BOOST trial mentioned above [16], the increase in mean LVEF was no longer significant at late follow-up at 18 months (5.9 vs 3.1 percentage points), suggesting that the benefit was limited to acceleration of LVEF recovery. Moreover, BMC infusion did not decrease the risk of adverse clinical events such as in-stent restenosis and arrhythmia. Notably, the following BOOST 2 clinical trial in which were enrolled 153 patients showed no significant increase in LVEF between BMC transfused and no transfused patients [30].

A series of studies including the HEBE trial [31], the REGENT trial [32], the MYSTAR trial [33] and the SWISS—AMI trial [34] that enrolled a total of 660 AMI patients showed only a modest or no significant improvement in global and regional LVEF after intracoronary BMC infusion.

In the ASTAMI trial [35, 36] 100 patients with AMI undergoing primary PCI were randomly assigned to intracoronary BMC infusion group or to control group. Serial imaging with multiple modalities (echocardiography, single photon emission computed tomography [SPECT] and cardiac magnetic resonance) were performed in each patient at baseline and at six months. No differences in LVEF or infarct size were observed between the two groups, suggesting that stem cell transplantation did not improve AMI outcome.

The ineffectiveness of stem cell transplantation in AMI patients was recently confirmed in CAREMI [37], MiHeart/AMI [38], BAMI [39] and Cardiovascular Cell Therapy Research Network (CCTRN) trials [40] including a total of 665 patients showing no improvement in LV remodelling and LVEF as well as in reducing the time to all-cause mortality.

Table 2 summarizes clinical trials showing uncertain results of stem cells transplantation after AMI.

**Table 2:** Clinical trials reporting uncertain benefit of stem cells transplantation after acute myocardial infarction

Author	Year	Reference	Trials	Cells source	N. patients	Mean % LVEF variation
Fuchs S	2003	[9]	–	BMC	10	=
Tse HF	2003	[6]	–	BMC	8	=
Janssens S	2006	[20]	–	BMC	67	< 1
Lunde K	2006	[29]	ASTAMI	BMC	50	> 0.6
Lunde K	2007	[30]	ASTAMI	BMC	100	=
Hirsch A	2008	[25]	HEBE	BMC	200	=
Tendera M	2009	[26]	REGENT	BMC	200	=
Gyongyosi	2009	[27]	MYSTAR	BMC	60	=
Surder D	2010	[28]	SWISS-AMI	BMC	192	=
Traverse JH	2012	[34]	TIME	BMC	120	=
Wollert KC	2017	[24]	BOOST-2	BMC	153	< 1
Fernandez-Avilés F	2018	[31]	CAREMI	Cardiac stem cells	49	=
Nicolau JC	2018	[32]	MiHeart/AMI	BMC	121	=
Mathur A	2020	[33]	BAMI	BMC	375	=
Total					1705	

*BMC* bone marrow-derived cells

## Discussion

Several studies have advanced the possibility that multipotent stem cells are capable to directly differentiate into cardiac myocytes and to regulate the crosstalk between endothelial cells, cytokines and cardiac cells, in order to favour coronary angiogenesis and substitute apoptotic dead cells after ischaemic myocardial damage. As in most drug development endeavours, there are phases of premature excitement followed by more realistic expectations. Cardiovascular regenerative/reparative medicine has passed the early phase of enthusiastic optimism and is building its foundations on more scientific evidence.

This review reports the largest number of blinded and unblended trials performed in the last two decades (2000–2020), enrolling a total of 3142 patients, aimed at evaluating the efficacy of stem cells transplantation after AMI.

In most trials, transplanted cells were bone marrow-derived cells and in a lower number of trials, transplanted cells were circulating progenitor cells, granulocyte-colony stimulating factor G-CSF mobilized peripheral blood stem cells, mesenchymal cells and allogenic cardiac stem cells.

Despite the large number of patients evaluated results demonstrated uncertain efficacy of stem cell transplantation on LVEF increase determined six months after AMI. In fact, 20 trials including 1437 patients showed a mean 7.2% LVEF increase while 14 trials including 1705 patients did not show any significant LVEF improvement.

One possible explanation of these elusive results could be the different amounts of infused cells. In this regard, studies focused on dose–response analysis after stem cells infusion demonstrate significant reduction in LVEF with higher doses (> 83 × 10^6^ cells) compared with lower doses (< 43 × 10^6^ cells) [41, 42]. Another possibility to explain the inconsistency of the results is the source of transplanted cells. In fact, it has been reported that haematopoietic stem cells (i.e. BMC, CPC and PSC) are able to migrate directly to sites of injury [43] but do not transdifferentiate into cardiac cells in ischaemic tissue [44] and have a deficient DNA repair system leading to an accelerated cell ageing [45]. Of interest, mesenchymal stem cells can differentiate into endothelial cells, smooth muscle cells and cardiac myocytes [46, 47] release cytokines that promote angiogenesis [48] prevent left ventricle fibrosis [49] and stimulate proliferation and differentiation of resident cardiac stem cells [50].

Moreover, the number of patients enrolled in many clinical trials was not sufficient to reach a statistical significance. For example, the BAMI trial planned to enrol about 3000 patients ended up with 375 patients. The low number of cases was insufficient to reach a statistical significance in mortality among patients and case–control group (7% and 3.82%, respectively) demonstrating impossible to test the hypothesis that bone marrow mononuclear cells therapy decreases mortality [39]*.*

Therefore, we suggest that the main goals for future trials will identify optimal dose and type of cells to infuse as well as determine the better transplantation protocol including number of enrolled patients, number of treatments and administration via (intravenous or intracoronary). 

With this perspective, a large clinical trial with the scope to achieve a consistent response regarding the efficacy of stem cells therapy to renew and repair myocardial damage after acute myocardial infarction is needed. According with the Editorial recently published by Bolli [51] “The most important thing in the post-BAMI era is to keep an open mind and let ourselves be guided by the evidence”. Moreover, one of the most important unanswered questions is which clinical conditions (diseases) should be prioritized to increase the probability of therapeutic success.
